# Urogenital myiasis – An atypical presentation

**DOI:** 10.4322/acr.2020.192

**Published:** 2020-12-14

**Authors:** Sayanti Paul, Purnima Upreti, Amrita Makhija, Ruchira Nautiyal

**Affiliations:** a Himalayan Institute of Medical Sciences, Department of Obstetrics and Gynaecology. Dehradun, Uttarakhand, India

**Keywords:** Female Uro-genital Diseases, Myiasis, Urethral Diseases, Vaginal Discharge

## Abstract

The infestation of the human body by maggots has been reported worldwide and occurs most commonly in people of lower socioeconomic status and poor personal hygiene. Urogenital is the rarest site of myiasis presentations. Here we report the case of a 20-year-old, sexually inactive female student who presented with a necrotic growth in the paraurethral region infested with numerous maggots. The lesion involved the urethra and the bladder base. She was treated with debridement and bladder irrigation. The cystoscopy and local examination performed 2 weeks after admission, confirmed the complete healing of the urogenital lesion. Managing this patient’s unique challenge was to assess the extent of the involvement and removal of all maggots from the deepest wound portion. The female internal and external urogenital myiasis is a very occasional and under-reported health hazard. Reporting such cases increases the public and physician awareness about the mode of presentation, right diagnosis, and available treatment options.

## INTRODUCTION

The term Myiasis is etymologically derived from the Greek word mya, which means fly. It was first used by Hope FW, in 1840^1^ while describing the infestation of live vertebrates by dipterous larvae. Human myiasis (HM) is reported worldwide, being more common among the lower socioeconomic status families of tropical countries. HM is classified in different types depending on the involved body part and the relation between the larvae and the host.^2^ Though different organs like the eye, skin, mouth, ear, and genitalia can be affected, the vulvovaginal infestation is rare. In this setting, peculiar predisposing factors include the lack of underwear use, cervical carcinoma, uterine prolapse, use of dirty clothing during menstruation, soiled clothing, and bedding. Here we report the case of urogenital myiasis in a 20-year-old well-educated girl with sound physical and mental health status.

## CASE REPORT

A 20-year-old unmarried college student, resident of the rural area of north India, attended the gynecology emergency complaining of excruciating pain and crawling sensation in the vulvar region for two days. She started with a foul-smelling vaginal discharge for 6 months, followed by a vulvar growth over the last month. Her menstrual cycle was regular, with no history of sexual exposure. There was no history of trauma, insect bite, chronic illness including tuberculosis, diabetes, immunosuppressive disease, or steroidal therapy. There was a history of reusing uncleaned clothes during menstruation and inadequate perineal hygiene, lack of underwear use, sitting in the fields for farming, using open-air uncleaned public toilets.

**Figure 1:**
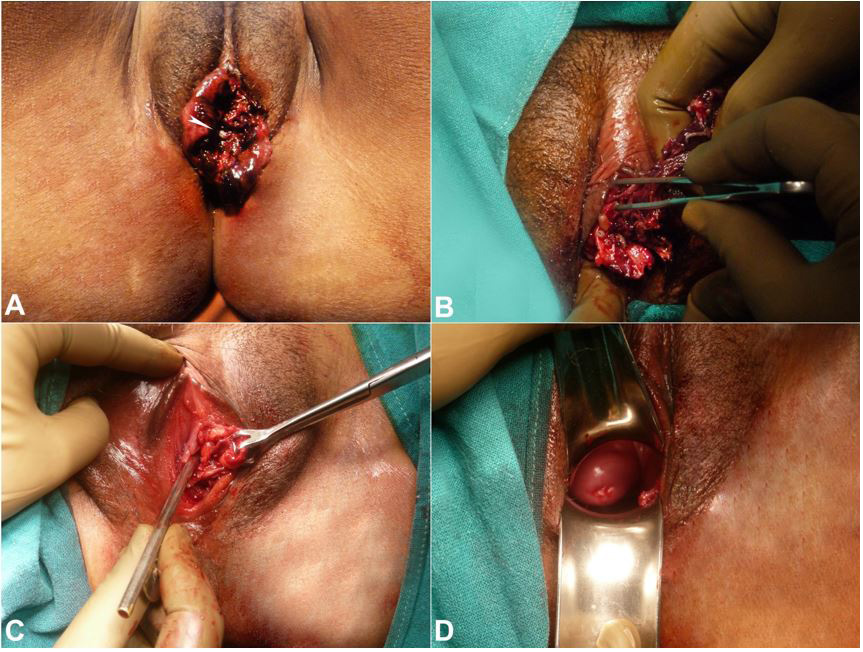
External view of the genitalia showing in **A –** Fungating vulvar mass before debridement with embedded maggot (arrow head); **B –** Debridement of necrotic vulvar mass with exposure of more deep-seated maggots; **C –** External view of vulva after debridement with the location of the paraurethral tunnel (location shown by Babcock’s forceps holding the lateral wall of the tunnel); **D –** speculum examination is showing the cervix and vagina free of any lesion.

Her physical examination was normal except for the external genitalia findings, where a fungating, necrotic, foul-smelling, and tender mass of size 4cm by 5cm centimeters involving labia minora and extending from clitoris to posterior fourchette (Figure 1A) was found.

Numerous maggots were seen crawling out of the mass. No significant lymphadenopathy was present. Baseline blood, urine, and serological investigations were normal, except for a mild leukocytosis. Visible maggots approximately 50-60 in number were removed. Although the vagina was not involved, a turpentine oil-soaked gauze was kept in the vagina to enhance the removal of maggots lying deep in the wound. Intravenous amoxycillin and clavulanic acid (1.2 g twice daily) and metronidazole (500 mg thrice daily) were started to prevent secondary bacterial infection. Examination under anesthesia (EUA) and cystoscopy was done the next day to determine the extent of the lesion. During EUA, labia minora was found to be replaced by fragile foul-smelling necrotic tissue, which was easily coming out during debridement, exposing more maggots (Figure 1B). Another 30-40 maggots removed with non-toothed forceps and a 4cm deep para-urethral tunnel was identified (Figure 1C) on the left side. The vagina, the cervix, and the perineum were healthy (Figure 1D).

On cystoscopy, a gross erosion and burrowing of the left lateral urethral wall and large clots embedded with maggots were identified within the bladder lumen. A bladder wash was undertaken, and a Foley’s catheter was kept in situ for 14 days. The patient was kept under observation for 5 days with regular perineal care to rule out any deep-seated retained maggots. A cystoscopy undertaken after two weeks documented a healed paraurethral lesion. On the discharge, the patient was advised to keep the vagina and vulva clean. The entomological study of the removed maggots could not be done due to the non-availability of trained personnel at our institution and the patient’s financial restraints.

## DISCUSSION

Larval infestation can be a result of obligatory or facultative parasites. When deposited in the human tissue, the Dipteran larvae feed and mature through three stages: first, second, and third instar. The third instar larvae will develop into a pupa, followed by an adult fly. Worldwide, *Cochliomyiahominivorax, Chrysomyabezziana,* and *W. magnifica* are the most common flies that cause obligatory human wound myiasis. Numerous species of *Muscidae, Calliphoridae*, and *Sarcophagidae* (filth flies) have been implicated in facultative wound myiasis.^3^ Anatomically cutaneous and cavitary myiasis are the commonest in humans. However, the condition remains under-reported due to cultural and social reasons. Urogenital myiasis constitutes only 0.7% of the human infestations with few cases reported in the literature.^4^ Depending on the location, it can be subdivided into external urogenital and internal urogenital myiasis. In a recent systematic review^5^ vagina was found to be the commonest site of female urogenital myiasis. Our case is an interesting and rare example of both external and internal urogenital myiasis involving the clitoris, labia minora with extension into the urinary bladder. Poor sanitation conditions, low socioeconomic status, poor mental or physical health, lack of satisfactory self-hygiene, cervical carcinoma, presence of a urethral stent, and sexually transmitted infections^6^ are prone factors leading to external urogenital myiasis. Drying of undergarments and homemade reusable menstrual clothes outside may serve as a source of deposition of fly eggs. In our patient, the persistent foul-smelling vaginal discharge, lack of underwear use, sitting in public toilets, and open grasslands together served as precipitating factors. Though the patient was a post-graduate student in college, the lack of public awareness and social stigma resulted in the delayed treatment-seeking, in this case.

General management protocol includes (i) mechanical elimination of all visible maggots, (ii) debridement of the necrotic tissue, (iii) irrigation with an antiseptic solution, and (iv) regular dressing of the wound till all maggots come out and healing starts. Agents like 15% chloroform in olive oil or any oil or topical treatment with 1% Ivermectin in propylene glycol solution can facilitate maggot removal. Another option is the local application of a thick layer of petroleum jelly, paraffin oil, or turpentine oil which suffocates the larvae and forces it to come out from the deep wound. In addition, endoscopic imaging may be needed in cases of internal urogenital myiasis to know the extent of involvement and removal of the maggots from cavities. Off label use of oral Ivermectin (150-200 mcg/kg body weight) is usually considered in special cases. Broad-spectrum antibiotic coverage is needed in case of extensive maggot infestation to prevent secondary bacterial infection. In our case, we used time to time local application of turpentine oil-soaked gauze to remove maggots and then went for wound debridement followed by cystoscopy in the operation room.

Peculiarities of our case were the young age of the patient, sexual inactivity, and higher level of education for the patient. Similar cases have been reported before by Cilla et al.^7^ in an 18-year-old girl, by Passos et al.^8^ in a 19-year-old patient with multiple sexual partners, by Rawat et al.^4^ in an 18-year-old unmarried sexually inactive patient, by Soulsby et al.^9^ in a 79-year-old lady, by Rasti et al.^10^ in a 26 yr old female, and by Mondal et al.^11^ in a 22-year-old lady on fourth week of puerperium. All these, including our case, emphasize the fact that no age, no reproductive status, and no education level can prevent this disease unless we increase the general public awareness by reporting such cases. Our case also illustrates the importance of raising public awareness regarding personal and menstrual hygiene in the form of using disposable sanitary pads, under-wears, and sanitary toilet facilities, especially in the rural population. The gynecologist should differentiate it from other chronic granulomatous infections of vulva, STDs and malignancies. Knowledge of the clinical entity and the close inspection of lesions are keys to the diagnosis.
